# Exploring ALK fusion in colorectal cancer: a case series and comprehensive analysis

**DOI:** 10.1038/s41698-024-00598-7

**Published:** 2024-05-13

**Authors:** Zi-Jing Li, William Pat Fong, Dong-Sheng Zhang, Hui-Yan Luo, Dong-Liang Chen, Yan-Yu Cai, Zhi-Gang Chen, Jian-Li Duan, Zi-Yao Huang, Yu-Ting Lu, Xiao-Xia Huang, Yu-Hong Li, De-Shen Wang

**Affiliations:** Department of Medical Oncology, State Key Laboratory of Oncology in South China, Guangdong Provincial Clinical Research Center for Cancer, Sun Yat-sen University Cancer Center, 510060 Guangzhou, P. R. China

**Keywords:** Molecular medicine, Targeted therapies, Cancer therapeutic resistance

## Abstract

Anaplastic lymphoma kinase (ALK) fusion-positive colorectal cancer (CRC) is a rare and chemotherapy-refractory subtype that lacks established and effective treatment strategies. Additionally, the efficacy and safety of ALK inhibitors (ALKi) in CRC remain undetermined. Herein, we examined a series of ALK-positive CRC patients who underwent various lines of ALKi treatment. Notably, we detected an ALK 1196M resistance mutation in a CRC patient who received multiple lines of chemotherapy and ALKi treatment. Importantly, we found that Brigatinib and Lorlatinib demonstrated some efficacy in managing this patient, although the observed effectiveness was not as pronounced as in non-small cell lung cancer cases. Furthermore, based on our preliminary analyses, we surmise that ALK-positive CRC patients are likely to exhibit inner resistance to Cetuximab. Taken together, our findings have important implications for the treatment of ALK-positive CRC patients.

## Introduction

The therapeutic landscape for colorectal cancer (CRC) has witnessed significant improvements over the years, owing to advancements in pathological staging, molecular stratification, microsatellite instability (MSI) status, and the identification of pivotal mutational drivers such as RAS and BRAF^1,2^. Nonetheless, CRC remains a heterogeneous disease that requires further subclassification, especially since molecular events play an essential role in determining treatment and survival outcomes. Anaplastic lymphoma kinase (ALK) fusions have been identified as a distinct molecular subtype and potent therapeutic target in several cancers, such as non-small cell lung cancer (NSCLC), accounting for ~4–6% of lung carcinomas^3^. ALK inhibitors (ALKi) have demonstrated remarkable benefits in ALK-positive NSCLC versus conventional chemotherapy regimens in recent years. Nevertheless, ALK fusions are exceptionally uncommon in CRC, with gene fusions comprising <1% of CRC cases^4^. Although ALK inhibitors have made substantial strides in the treatment of ALK-positive NSCLC, their effectiveness and safety in the context of CRC remain underreported.

To that end, the present study aims to offer valuable insights into the clinical, molecular, and pathological attributes of ALK-positive CRC. Furthermore, we assessed the efficacy and investigated acquired resistance associated with ALKi.

## Results

### Clinical and molecular characteristics of ALK-positive CRC patients

A comprehensive analysis of ALK-positive CRC cases was performed by consolidating data from Sun Yat-sen University Cancer Center (SYSUCC; *N* = 6) spanning from December 2020 to January 2023, along with information extracted from previously published cases (*N* = 9)^5–9^. Patient characteristics of all included patients (*N* = 15) are detailed in Supplementary Table 1. The median age at diagnosis for the cohort was 53 years (ranging from 43 to 87 years), with female patients comprising 53% of the cases. Notably, most patients (*N* = 11, 73.3%) presented with right-sided primary colon cancer, with 80% (*N* = 12) exhibiting adenocarcinoma histology. The most prevalent type of ALK fusion was EML4-ALK, accounting for 40% of patients. Of note, Serine/arginine-rich splicing factor 7 (SRSF7) was identified as a new fusion partner for ALK in Case 6 (C6). Microsatellite stable (MSS) status was observed in 12 patients, while one patient displayed MSI-H, and the status of two patients is unknown. Over half of the included patients (*N* = 9) had multiple metastatic sites, with data missing for five patients. Three patients previously received Cetuximab, while four were treated with Bevacizumab. In terms of ALKi treatment, three patients (27%) were treated with Alectinib, two patients (18%) with Ensartinib, one patient (9%) with Entrectinib, four patients (37%) with Crizotinib, and one patient (9%) with Ceritinib. After disease progression under first-line ALKi, five patients (46%) received subsequent lines of ALKi treatment. Alectinib was administered after Crizotinib in three patients, Brigatinib was utilized following Ensartinib in one patient, and Ceritinib was introduced after the failure of upfront Alectinib in one patient. Interestingly, all included ALK fusion-positive CRC patients did not harbor activated mutations in RAS/RAF genes, which are the predominant driver gene mutations in CRC. Meanwhile, Case 3 (C3) exhibited ERBB2 amplification, FGFR1 amplification, and CDK12 amplification, while Case 5 (C5) harbored an RSPO2 fusion.

### Activity and efficacy of ALKi

Table 1 provides an overview of the patients who underwent ALKi therapy. A total of 11 patients received ALKi treatment, including two patients from the SYSUCC cohort and nine patients from published studies. The median progression-free survival (PFS) for the first ALKi treatment was 4.5 months (Supplementary Fig. 2). Among these patients, two (18%) achieved a complete response, while five (45%) achieved a partial response (PR). The objective response rate (ORR) was 63%, and three patients (27%) reached stable disease (SD), resulting in a disease control rate (DCR) of 90%. Only one patient (9%) experienced disease progression during the initial evaluation.

**Table 1:** Clinical and molecular characteristics of patients who underwent ALKi therapy

Patient ID	Age	Gender	Primary tumor site	Histology	Stage at diagnosis	Surgery/locoregional treatment	Lines of systemic treatment before ALKi	ALK fusion partner	MSI/MSS status	Other NGS data	First-Line ALKi agent	Best tumor response	First-Line ALKi PFS (months)	Further ALKi agent (PFS[months])	Reference
P1	53	Female	Right colon	Adenocarcinoma	IV	Surgery	1	CAD-ALK	NA	TP53 p.R248W	Entrectinib	CR	4.5	None	^5^
P2	70	Female	Right colon	Adenocarcinoma	IV	None	2	EML4-ALK	MSS	TP53 R157H、AKT1、ANNKRD11、BRAF、DNMT3A、FLT4、GABRA6、PTPRT、RBM10、SLX4	Crizotinib	PR	3	Alectinib (0.5 months), Lorlatinib (11.5 months)	^6^
P3	87	Female	Right colon	Adenocarcinoma	IV	Surgery	3	STRN-ALK	MSI	KRAS R164Q、STK11、TP53、AEID1A、BCOR、CDH2、MLL2、MLL3、PAX5、RAD50	Ceritinib	PR	9	None	^7^
P4	53	Female	Right colon	Adenocarcinoma	IV	None	2	CAD-ALK	MSS	ALK 1196Q、TP53 R273C	Alectinib	CR	3.8	None	^8^
P5	46	Male	Rectum	Adenocarcinoma	IV	None	2	EML4-ALK	MSS	KDR, SMAD4, APC, TP53	Crizotinib	PD	2.3	None	^8^
P6	45	Male	Right colon	Adenocarcinoma	IV	None	2	EML4-ALK	MSS	TP53 splice site, APC truncating mutation	Crizotinib	PR	9.1	Alectinib (NA)	^8^
P7	51	Female	Right colon	Adenocarcinoma	IV	None	2	CAD-ALK	MSS	TP53	Crizotinib	SD	3.7	Alectinib (NA)	^8^
P8	50	Female	Right colon	Adenocarcinoma	IV	Surgery	2	CAD-ALK	NA		Ensartinib	PR	4.6	None	^8^
P9	67	Male	Left colon	Adenocarcinoma	IV	Surgery	1	STRN-ALK	MSS	TP53, PIK3CG, PTPRS, PTPRT, MYC	Alectinib	SD	14	Ceritinib (NA)	^8^
C5	43	Male	Right colon	Adenocarcinoma	III	Surgery	2	EML4-ALK	MSS	PIK3R2 p.R717H;TP53 p.L43*;LRP1B p.K1100R;WIPF1 p.G216R；NUDCD1-RSPO2Fusion	Ensartinib	PR	5.8	Brigatinib (3 months); Lorlatinib (2 months)	–
C6	77	Female	Left colon	Signet-ring cell	IV	None	1	SRSF7-ALK	MSS	MSH6 p.F1088fs；TP53 c.75-1G>C	Alectinib	SD	1.9	None	–

*ALKi* ALK inhibitor, *MSS* microsatellite stability, *MSI* microsatellite instability, *CR* complete response, *PR* partial response, *PD* progressive disease, *SD* stable disease, *NA* not available.

Among the two patients who received second-line ALKi treatment, P2 experienced progressive disease (PD) after 0.5 months of Alectinib treatment. Subsequently, this patient underwent Lorlatinib treatment for 11.5 months before passing away. Meanwhile, C5 received Brigatinib and Lorlatinib as a third- and fourth-line of ALKi treatment, respectively. As of the cutoff date, all patients had experienced PD following first-line ALKi treatment, with a total of 11 patients (85%) succumbing to the disease.

The swimmer plot in Supplementary Fig. 3 further illustrates the clinical treatment received by the six patients: C1, C2, C3, C5, P2, and P4. Among these, C1, C2, and C3 received Bevacizumab, while C3, C5, P2, and P4 were treated with Cetuximab. Given that the addition of Cetuximab for ALK fusion-positive CRC patients yielded suboptimal outcomes, we further compared the PFS of different targeted therapies regardless of the treatment line. As shown in Supplementary Fig. 4, the PFS of patients who received Cetuximab was significantly worse than those who received Bevacizumab (hazard ratio [HR] 4.41, 95% CI [0.78, 24.82], log-rank *P* = 0.021).

### Representative case of ALK fusion-positive CRC receiving ALKi sequential strategy

**Fig. 1:**
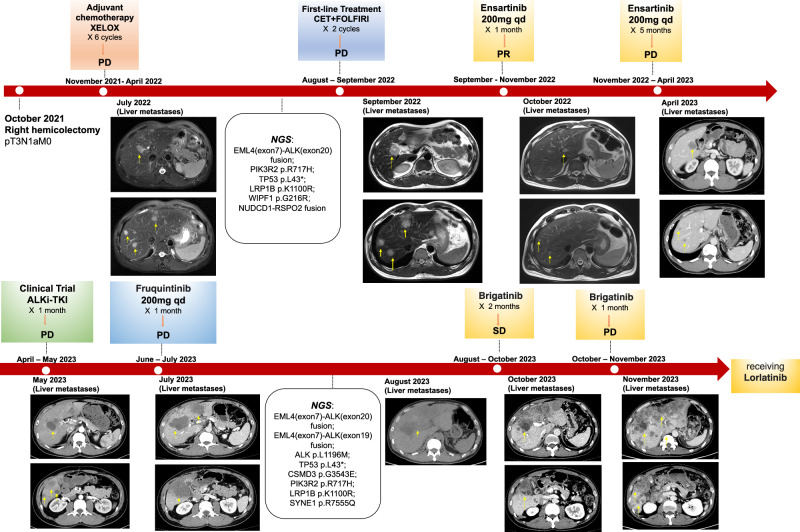
Representative clinical history of a patient who received multiple lines of ALKi treatment. XELOX capecitabine and oxaliplatin, PD progressive disease, PR partial response, SD stable disease, NGS next-generation sequencing.

Figure 1 presents a detailed timeline of the clinical history of a representative case, C5, a 43-year-old male diagnosed with advanced CRC. The patient developed liver metastases following adjuvant XELOX chemotherapy. Subsequent NGS testing of the primary tumor revealed the presence of EML4 (exon7)-ALK (exon20) fusion, which is notably distinct from EML4-ALK V1 (exon 13 of EML4 fused to exon 20 of ALK) or V3 (exon 20 of EML4 fused to exon 20 of ALK) variants. Besides, NGS testing further revealed that the patient harbored NUDCD1-RSPO2 fusion, while FGFR gene fusion was not detected. Given his RAS/RAF wild-type status, Cetuximab plus FOLFIRI was initiated. However, rapid progression was observed after just two cycles of chemotherapy. Next, second-generation ALKi, Ensartinib, was administered, and the patient achieved PR status within one month. Regrettably, after 5 months, his liver metastases showed progression, and the patient opted for a clinical trial involving a third-generation ALKi, which also proved ineffective. The patient was then given Fruquintinib as part of the standard third-line treatment regimen for CRC. Nevertheless, tumor progression was detected, and a liver biopsy was recommended, considering the possibility of ALK inhibitor resistance. A biopsy of the liver metastases and NGS testing revealed the presence of the ALK L1196M mutation, often referred to as a gatekeeper mutation, in addition to EML4 (exon7)-ALK (exon19) fusion. Based upon previous studies that showed that Brigatinib and Lorlatinib are effective against ALK L1196M resistance mutations^10,11^, Brigatinib was initiated, and the patient achieved good disease control and experienced relief from abdominal pain and a significant reduction in serum CEA levels after receiving Brigatinib for one month. However, after 3 months of initiating Brigatinib treatment, disease progression was observed, accompanied by a significant elevation in CEA levels. Subsequently, the patient was switched to Lorlatinib and has yet to exhibit disease progression to date.

## Discussion

While the clinical characteristics of ALK-fusion gastrointestinal tumors have been previously documented, there is a dearth of data regarding the efficacy of ALKi in patients with ALK-positive CRC due to its rarity in colorectal malignancies. Herein, we conducted a comprehensive analysis encompassing clinical, pathological, and molecular characteristics, along with an in-depth evaluation of treatment efficacy in patients with ALK fusion-positive CRC. Notably, our study identified an instance of acquired resistance to second-generation ALKi in a patient who underwent multiple lines of ALKi therapy.

Prior studies had suggested that ALK-positive CRC was often associated with MSI status^9,12^. However, our analysis revealed that most CRC patients with ALK fusion exhibited microsatellite stability (MSS) status. It is worth noting that gene fusions are recognized as a significant mechanism of oncogenic activation and are typically mutually exclusive with other oncogenic mutations^12^. Consistently, we observed that most ALK-positive CRC patients were mutually exclusive with common driver mutations such as RAS/RAF. However, we identified one patient who harbored ERBB2 amplification at the same time, which could suggest the possibility of additional underlying mechanisms that merit further investigation.

Herein, we identified a previously undocumented ALK fusion partner, SRSF7. Previous genome-wide CRISPR/Cas9 screenings have highlighted SRSF7 as a critical gene that regulates cell growth and viability in multiple cancer cell lines^13^. Furthermore, research has revealed that SRSF7 functions as an oncogene and is overexpressed in various cancers. Thus, the interplay between SRSF7 and ALK fusion warrants further exploration.

Additionally, we reveal that ALK fusion-positive CRC patients might most likely exhibit inherent resistance to Cetuximab, which is in line with the outcomes observed in previous studies^14–16^. This aligns with the recognition of ALK fusion as a potentially positive and hyperselective marker for Cetuximab resistance, as indicated in the PARADIGM study^17^. These results underscore the significance of excluding ALK fusion-positive patients when considering Cetuximab as a first-line treatment for CRC. Previous studies^18^ have established that ALK fusion proteins engage with a complex network of downstream proteins via various downstream pathways, including JAK/STAT, PI3K/AKT, and MEK/ERK, to drive aberrant proliferation and survival. Consequently, ALK fusion proteins may circumvent anti-EGFR inhibition to activate downstream components of EGFR, thereby promoting cancer cell proliferation and survival, which could explain the possible mechanism of inner resistance to Cetuximab observed in our patients. Moreover, certain tyrosine kinases, including ALK, have demonstrated intrinsic resistance to Cetuximab in CRC cell lines^19^. Nevertheless, given the rarity of ALK fusions in CRC, larger-scale clinical studies are warranted to validate the influence of ALK fusions on the effectiveness of anti-EGFR inhibitors and to determine whether Bevacizumab could instead yield superior treatment outcomes.

In NSCLC, the echinoderm microtubule-associated protein-like 4 gene (EML4) is the most common fusion partner of ALK, giving rise to over 15 distinct EML4-ALK fusion variants. This pattern is consistent with what has been reported in CRC, where EML4-ALK fusion is the most prevalent type of ALK fusion. Interestingly, we also observed an uncommon EML4-ALK fusion variant, EML4 (exon7)- ALK (exon19) fusion, in a patient who received multiple lines of chemotherapy and ALKi. While this specific variant has been documented in both NSCLC^20^ and CRC^21^, its significance remains unknown. Moreover, given that it was detected during disease progression, further research is needed to elucidate the role of EML4 (exon7)-ALK (exon19) in acquired resistance to ALKi.

Acquired resistance to ALKi primarily arises from mutations in the target gene, rendering the encoded tyrosine kinases resistant to inhibition. The first identified resistance point mutations were ALK C1156Y and ALK L1196M. Given the response exhibited by our representative case, we surmise that Brigatinib can suppress tumor clones carrying secondary resistance mutations to Ensartinib since Brigatinib could overcome the ALK L1196M mutation in NSCLC. Herein, we detected an ALK L1196M mutation in a CRC patient treated with second-generation ALKi. Although Brigatinib initially showed good disease control and significant symptom alleviation at first, the patient experienced disease progression three months later. Notably, the median PFS for all included ALK-positive CRC patients receiving ALKi therapy for the first time was 4.5 months. In contrast, individuals with ALK-positive NSCLC who received first-generation ALK inhibitors as their initial treatment have a reported median PFS of 10.9 months^22^, suggesting potentially lower efficacy of ALK inhibitors in ALK-positive CRC compared to NSCLC. Additionally, the patient’s short-term response to Brigatinib may also be attributed, in part, to their history of multiple chemotherapy lines and prior ALK inhibitor therapy.

Biomarker-driven therapy has been gaining prominence in the treatment paradigm for CRC, with several regulatory-approved drugs such as larotrectinib for NTRK fusions, pembrolizumab and dostarlimab for dMMR/MSI-H, and selpercatinib for RET fusion^23^. This underscores the pressing need for further clinical trials for drugs targeting ALK fusion in CRC.

Collectively, we identified an ALK L1196M mutation in an ALK-positive CRC patient, while in another patient, we uncovered an unreported ALK fusion partner, SRSF7. Importantly, in terms of translational significance, we surmise that Brigatinib and Lorlatinib could be effective in managing CRC patients harboring ALK L1196M resistance mutation. Nevertheless, larger-scale studies with overall survival data are imperative to validate our findings.

## Methods

### Patients population

The inclusion criteria were CRC patients diagnosed with ALK fusion. Patient-related information, encompassing clinical characteristics, primary tumor site, pathological features, ALK fusion partners, microsatellite stability status, baseline NGS, mutational data, as well as details on clinical treatments received. Importantly, all patients underwent NGS for ALK fusion detection. This retrospective study was conducted under the ethical approval granted by the Sun Yat-sen University Ethics Committee (Approval ID: SL-B2023-496-01) and adhered to the principles outlined in the Declaration of Helsinki. No personal identifiers were used in the analysis. NGS testing for the SYSUCC cohort was conducted after all participants provided signed and written informed consent. Given the retrospective nature of the present study, the ethics committee of SYSUCC waived the requirement for additional informed consent for data analysis.

### NGS test

Patients in the SYSUCC cohort subjected to NGS analysis were required to have >10% of tumor cells identified by IHC. Patients were subjected to a wide panel genomic sequencing (pan-cancer 1326-gene panel) for simultaneous detection of MSI status and mutations in 1326 cancer-related genes, including KRAS, NRAS, BRAF, HER2, TP53, and ALK, among others.

### Outcome assessment

Progression-free survival was defined as the time from the start of the ALKi agent or targeted therapy to PD or death, whichever occurred first. Disease response was evaluated in accordance with the RECIST 1.1 criteria. ORR was defined as the proportion of patients who achieved CR or partial response PR to ALK inhibitors. Additionally, DCR denoted the percentage of patients who reached either CR, PR, or SD. The data cutoff date for these assessments was January 13, 2024.

### Statistical analyses

Categorical variables were expressed using frequency (*n*) and percentage (%) with a 95% CI where applicable. The median PFS and its 95% CI were calculated using the Kaplan–Meier method. The log-rank test was used to compare the treatment groups in terms of PFS and a Cox proportional hazards model estimated hazard ratios (HRs) with 95% CIs. Visual representations, such as the gene mutation overview, survival curve, and swimmer plot, were generated using R. All statistical analyses and graphical representations were conducted using R software version 4.2.2, and statistical significance was defined as a *P* value < 0.05.

### Reporting summary

Further information on research design is available in the Nature Research Reporting Summary linked to this article.

### Supplementary information

REPORTING SUMMARY

Supplemental material

## Data Availability

Separate patient consent for depositing genetic data was not obtained; therefore, we were unable to upload these data to a public repository in accordance with privacy laws in China. However, data reported in this article will be available upon reasonable request from the corresponding authors, subject to compliance with ethics and privacy laws. Alternatively, researchers may request access through our database administrator, the relevant data is stored at Sun Yat-sen University’s research platform (https://rdd.sysu.edu.cn/, Deposit ID: 2402020007). For access, please email yjsinfo@mail.sysu.edu.cn by submitting a formal request.

## Code availability

The Complexheatmap package in R was used to create the oncoplot of the molecular information, and the survival and survminer packages were used to construct the Kaplan–Meier survival curves. The swimmer package was utilized to depict treatment received using a swimmer plot.

## References

[1] Ye L-F (2022). Monitoring tumour resistance to the BRAF inhibitor combination regimen in colorectal cancer patients via circulating tumour DNA. Drug Resist. Updat..

[2] Wang Z-X (2021). Temporal change in treatment patterns of metastatic colorectal cancer and its association with patient survival: a Retrospective Cohort Study Based on an Intelligent Big-Data Platform. Engineering.

[3] Soda M (2007). Identification of the transforming EML4-ALK fusion gene in non-small-cell lung cancer. Nature.

[4] Delaye M (2022). Rational testing for gene fusion in colorectal cancer: MSI and RAS-BRAF wild-type metastatic colorectal cancer as target population for systematic screening. Eur. J. Cancer.

[5] Siravegna G (2017). Tracking a CAD-ALK gene rearrangement in urine and blood of a colorectal cancer patient treated with an ALK inhibitor. Ann. Oncol..

[6] He, X. et al. Clinical responses to Crizotinib, Alectinib, and Lorlatinib in a metastatic colorectal carcinoma patient with ALK gene rearrangement: a Case Report. *JCO Precis. Oncol.* **5**, 10.1200/PO.20.00534 (2021). 10.1200/PO.20.00534 PMC8140796 34036227

[7] Yakirevich E (2016). Oncogenic ALK fusion in rare and aggressive subtype of colorectal adenocarcinoma as a potential therapeutic target. Clin. Cancer Res..

[8] Ambrosini M (2022). ALK inhibitors in patients with ALK fusion-positive GI cancers: an International Data Set and a Molecular Case Series. JCO Precis. Oncol..

[9] Singh H (2021). Molecular characterization and therapeutic targeting of colorectal cancers harboring receptor tyrosine kinase fusions. Clin. Cancer Res..

[10] Yoda S (2018). Sequential ALK inhibitors can select for lorlatinib-resistant compound ALK mutations in ALK-positive lung cancer. Cancer Discov..

[11] Zhang S (2016). The potent ALK inhibitor brigatinib (AP26113) Overcomes mechanisms of resistance to first- and second-generation ALK inhibitors in preclinical models. Clin. Cancer Res..

[12] Pietrantonio, F. et al. ALK, ROS1, and NTRK rearrangements in metastatic colorectal cancer. *J. Natl Cancer Inst.* **109**, 10.1093/jnci/djx089 (2017). 10.1093/jnci/djx089 29370427

[13] Behan FM (2019). Prioritization of cancer therapeutic targets using CRISPR-Cas9 screens. Nature.

[14] Cremolini C (2017). Negative hyper-selection of metastatic colorectal cancer patients for anti-EGFR monoclonal antibodies: the PRESSING case-control study. Ann. Oncol..

[15] Morano F (2019). Negative hyperselection of patients with RAS and BRAF wild-type metastatic colorectal cancer who received panitumumab-based maintenance therapy. J. Clin. Oncol..

[16] Nussinov R, Tsai C-J, Jang H (2021). Anticancer drug resistance: an update and perspective. Drug Resist Updat.

[17] Yoshino T (2017). Rationale for and design of the PARADIGM study: Randomized Phase III study of mFOLFOX6 plus bevacizumab or panitumumab in chemotherapy-naïve patients with RAS (KRAS/NRAS) wild-type, metastatic colorectal cancer. Clin. Colorectal Cancer.

[18] Lin JJ, Riely GJ, Shaw AT (2017). Targeting ALK: precision medicine takes on drug resistance. Cancer Discov..

[19] Medico E (2015). The molecular landscape of colorectal cancer cell lines unveils clinically actionable kinase targets. Nat. Commun..

[20] Penzel R, Schirmacher P, Warth A (2012). A novel EML4-ALK variant: exon 6 of EML4 fused to exon 19 of ALK. J. Thorac. Oncol..

[21] Okano S (2023). Tyrosine kinase alterations in colorectal cancer with emphasis on the distinct clinicopathological characteristics. Histopathology.

[22] Solomon BJ (2014). First-line Crizotinib versus chemotherapy in ALK-positive lung cancer. N. Engl. J. Med..

[23] Bhamidipati D, Subbiah V (2023). Impact of tissue-agnostic approvals for patients with gastrointestinal malignancies. Trends Cancer.

