# Supplementary material for: Exploring ALK fusion in colorectal cancer: a case series and comprehensive analysis
Source: NPJ Precis Oncol. 2024 May 13;8:100. doi: 10.1038/s41698-024-00598-7 (PMC11091044; doi:10.1038/s41698-024-00598-7)
Supplement: Supplementary file 2 — Supplemental material [file 41698_2024_598_MOESM2_ESM.pdf]

## Supplementary Files

**Supplementary Table 1. Cumulative Frequencies of Patients' Characteristics**

| Characteristics | Patients, No. (%) | Characteristics | Patients, No. (%) |
|---|---|---|---|
| Age, years | | Peritoneal metastases | |
| Median(range) | 53(43-87) | Yes | 3(20) |
| Sex | | No | 7(47) |
| Male | 7(47) | NA | 5(33) |
| Female | 8(53) | Brain metastases | |
| Primary tumor site | | Yes | 1(7) |
| Left colon | 4(27) | No | 9(60) |
| Right colon | 11(73) | NA | 5(33) |
| Histology | | MSI/MSS status | |
| Adenocarcinoma | 14(93) | MSI-H | 1(7) |
| Signet-ring cell | 1(7) | MSI-L/MSS | 12(80) |
| Stage at diagnosis | | NA | 2(13) |
| I-II | 0(0) | Fusion partners | |
| III | 1(7) | EML4 | 6(40) |
| IV | 14(93) | STRN | 3(20) |
| Metastasis presentation | | CAD | 4(26) |
| Synchronous | 7(47) | PPM1G | 1(7) |
| Metachronous | 0(0) | SRSF7 | 1(7) |
| NA | 8(53) | Received ALKi | |
| No. of metastatic sites | | Yes | 11(73) |
| 1 | 1(7) | No | 4(27) |
| ≥2 | 9(60) | First line ALKi (agent) | |
| NA | 5(33) | Entrectinib | 1(9) |
| Liver metastases | | Crizotinib | 4(37) |
| Yes | 5(33) | Ceritinib | 1(9) |
| No | 5(33) | Alectinib | 3(27) |
| NA | 5(34) | Ensartinib | 2(18) |
| Nodal metastases | | Further lines of ALKi | |
| Yes | 7(47) | Yes | 5(46) |
| No | 3(20) | No | 6(54) |
| NA | 5(33) | Further lines of ALKi (agent) | |
| Lung metastases | | Alectinib | 4(80) |
| Yes | 2(13) | Ceritinib | 1(20) |
| No | 8(54) | RAS/RAF status | |
| NA | 5(33) | Wild | 15(100) |
| Targeted therapy | | Mutant | 0(0) |
| Cetuximab | 4(27) | | |
| Bevacizumab | 3(20) | | |

| | |
|---|---|
| No targeted therapy | 3(20) |
| NA | 5(33) |

**Supplementary Table 1. Cumulative Frequencies of Patients' Characteristics.** The table comprised six patients from the Sun Yat-sen University Cancer Center (SYSUCC) cohort and nine from previously published cases. Synchronous metastases is defined as presence of metastases within 6 months after primary tumor diagnosis or following primary tumor resection. Metachronous metastases is defined as metastases diagnosed more than 6 months after primary tumor diagnosis. Abbreviations: MSI-H, microsatellite instability high; MSI-L, microsatellite instability low; MSS, microsatellite stable; NA, not available; ALKi, ALK inhibitor.

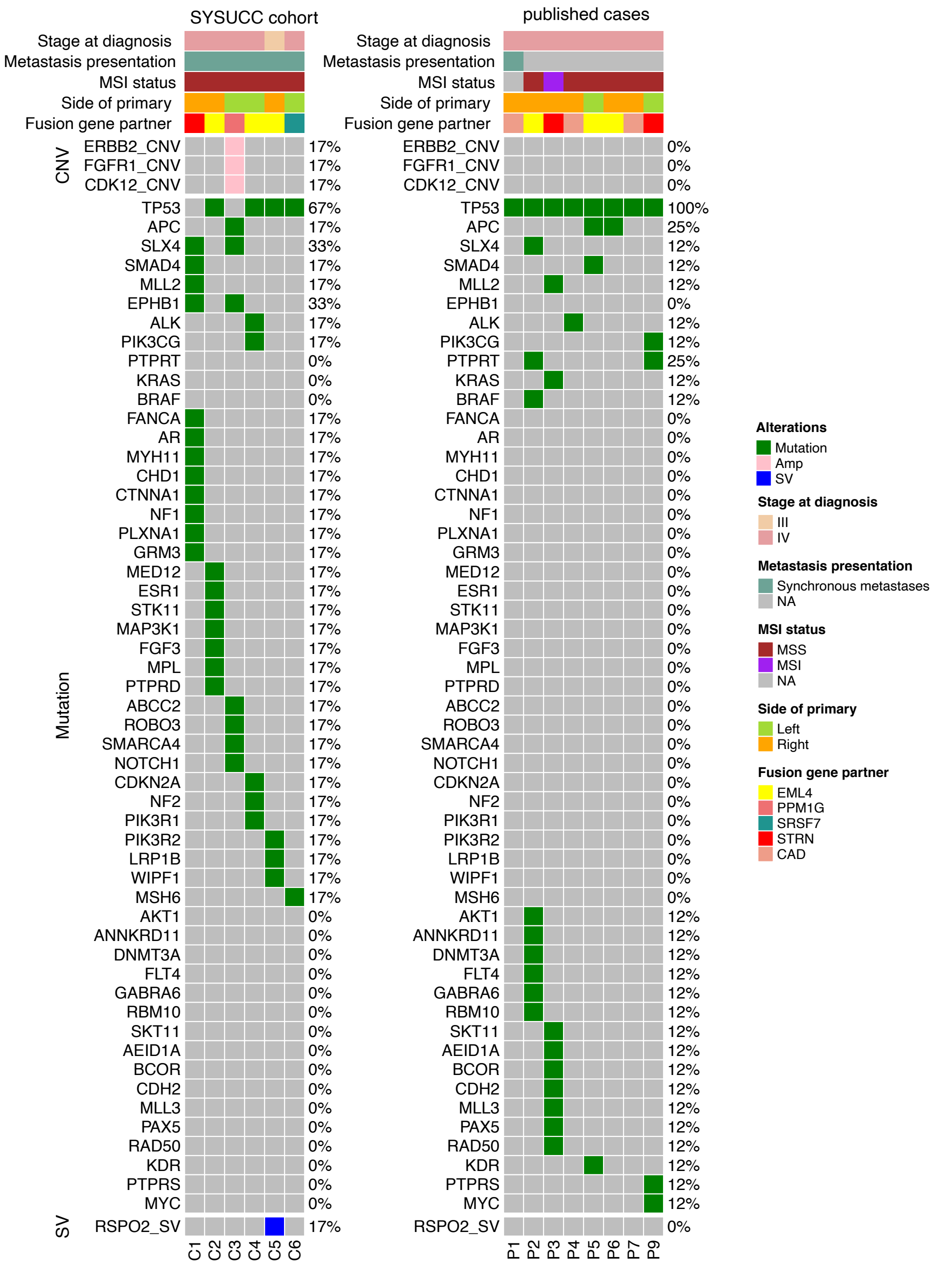
SYSUCC cohort
published cases
Stage at diagnosis
Metastasis presentation
MSI status
Side of primary
Fusion gene partner
CNV
Mutation
SV
Alterations
Mutation
Amp
SV
III
IV
Synchronous metastases
NA
MSS
MSI
Left
Right
EML4
PPM1G
SRSF7
STRN
CAD
C1 C2 C3 C4 C5 C6
P1 P2 P3 P4 P5 P6 P7 P9
ERBB2_CNV 17% 0%
FGFR1_CNV 17% 0%
CDK12_CNV 17% 0%
TP53 67% 100%
APC 17% 25%
SLX4 33% 12%
SMAD4 17% 12%
MLL2 17% 12%
EPHB1 33% 0%
ALK 17% 12%
PIK3CG 17% 12%
PTPRT 0% 25%
KRAS 0% 12%
BRAF 0% 12%
FANCA 17% 0%
AR 17% 0%
MYH11 17% 0%
CHD1 17% 0%
CTNNA1 17% 0%
NF1 17% 0%
PLXNA1 17% 0%
GRM3 17% 0%
MED12 17% 0%
ESR1 17% 0%
STK11 17% 0%
MAP3K1 17% 0%
FGF3 17% 0%
MPL 17% 0%
PTPRD 17% 0%
ABCC2 17% 0%
ROBO3 17% 0%
SMARCA4 17% 0%
NOTCH1 17% 0%
CDKN2A 17% 0%
NF2 17% 0%
PIK3R1 17% 0%
PIK3R2 17% 0%
LRP1B 17% 0%
WIPF1 17% 0%
MSH6 17% 0%
AKT1 0% 12%
ANNKRD11 0% 12%
DNMT3A 0% 12%
FLT4 0% 12%
GABRA6 0% 12%
RBM10 0% 12%
SKT11 0% 12%
AEID1A 0% 12%
BCOR 0% 12%
CDH2 0% 12%
MLL3 0% 12%
PAX5 0% 12%
RAD50 0% 12%
KDR 0% 12%
PTPRS 0% 12%
MYC 0% 12%
RSPO2_SV 17% 0%

**Supplementary Figure 1.** Oncoplot for mutation, amplification, and structural variants of patients harboring ALK fusion in the SYSUCC cohort and previously published cases. The Oncoplot provides an overview of mutations in particular genes (rows) affecting each patient (columns). Patient identification is at the bottom of the plot. Abbreviations: MSS, microsatellite stable; MSI, microsatellite instability; NA, not available; Amp, amplification; SV, structural variants.

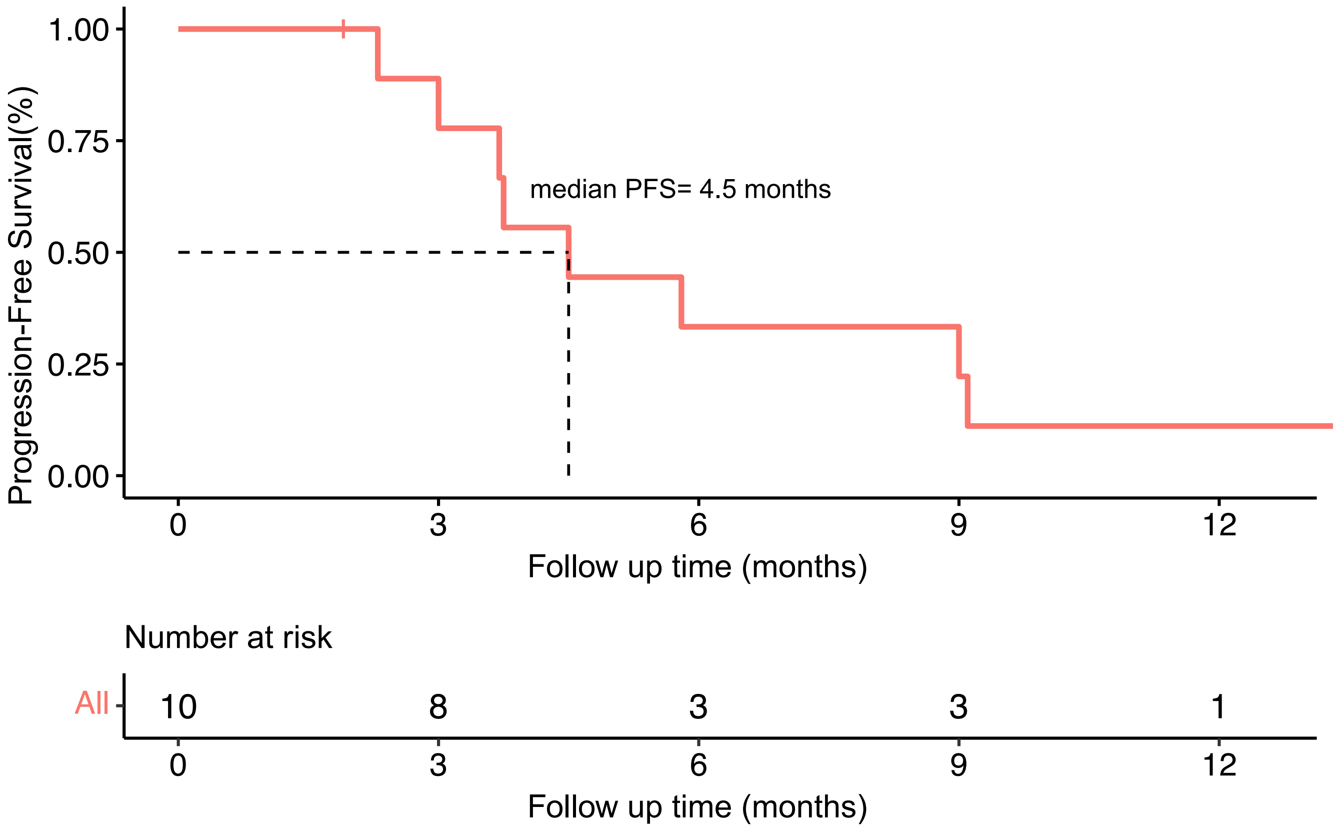


**Supplementary Figure 2. PFS of the 11 patients that received first-line ALK inhibitor.** Abbreviation: PFS, progression-free survival.

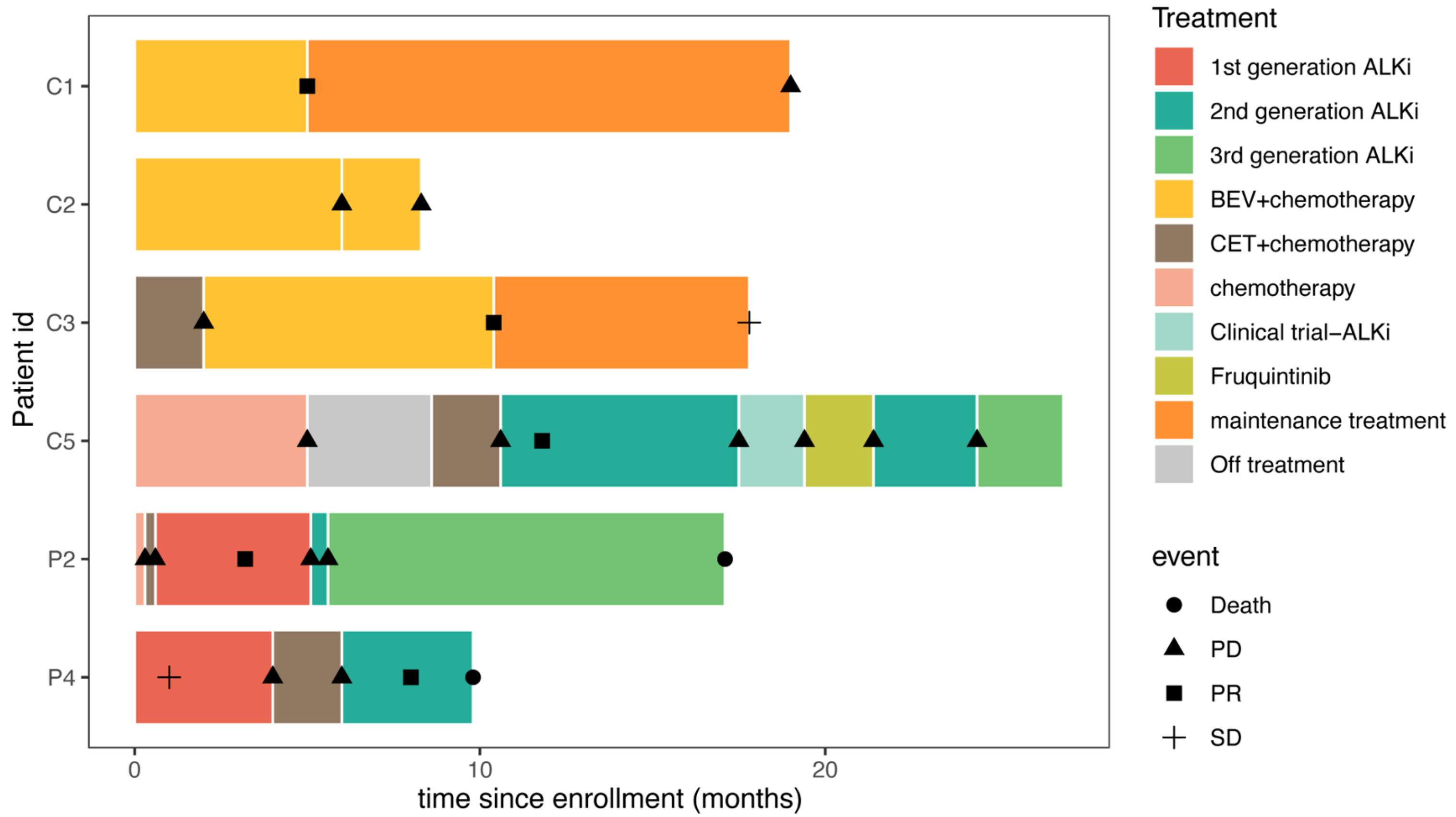


**Supplementary Figure 3. The swimmer plot of patients that received targeted therapy.** Maintenance treatment consisted of bevacizumab plus capecitabine. Patients that received targeted therapy include C1, C2, C3, C5, P2 and P4. Abbreviation: ALKi, ALK inhibitor; BEV, Bevacizumab; CET, Cetuximab; PR, partial response; PD, progressive disease; SD, stable disease.

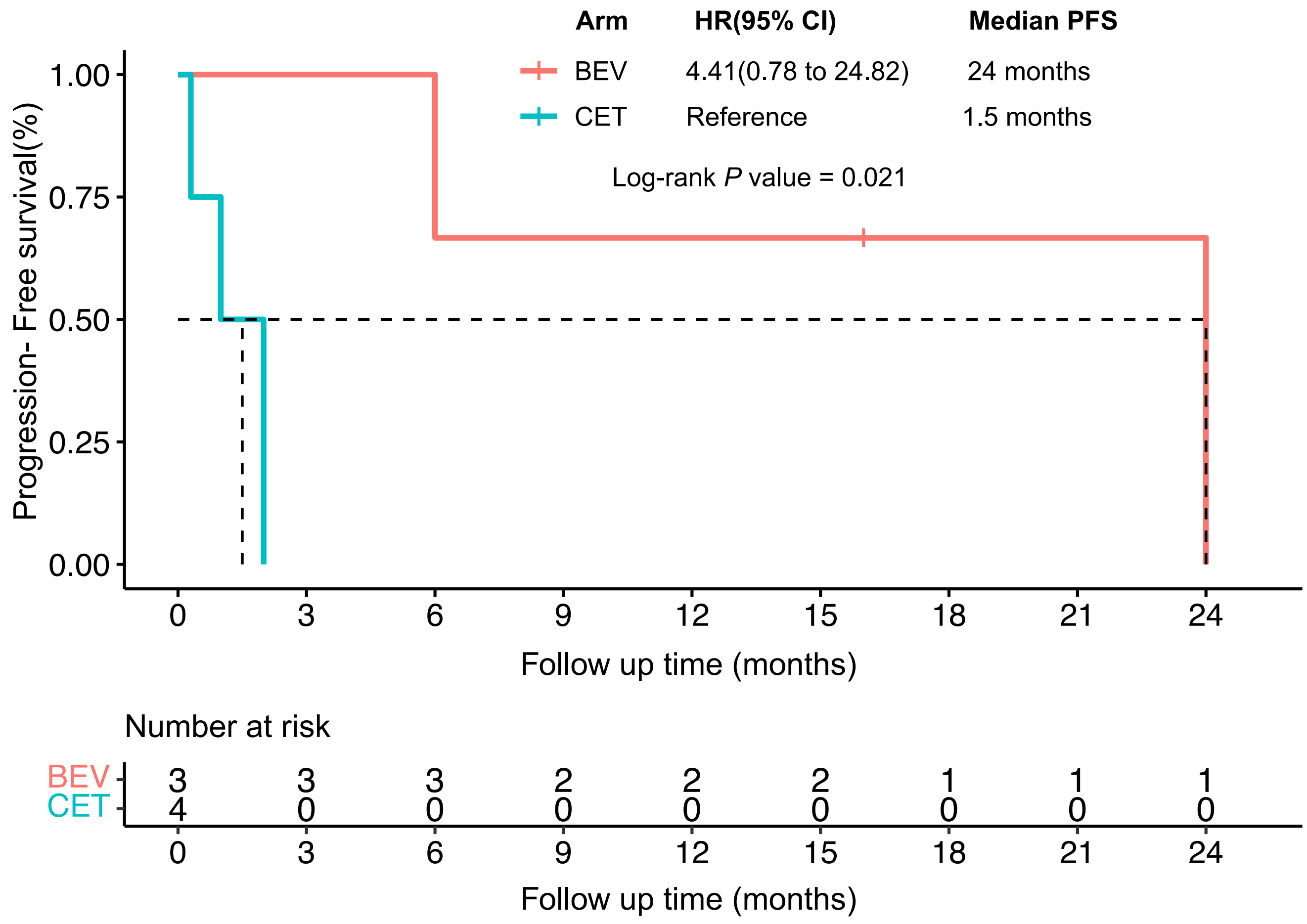


**Supplementary Figure 4. The progression-free survival (PFS) of patients who received targeted therapy.** PFS was defined as from the start of the targeted therapy to PD or death, whichever occurred first. Patients included in this plot are C1, C2, C3, C5, P2 and P4. Abbreviation: BEV, Bevacizumab; CET, Cetuximab. HR, hazard ratio; CI, confidence interval.
